# The intestinal phosphate transporter NaPi-IIb (Slc34a2) is required to protect bone during dietary phosphate restriction

**DOI:** 10.1038/s41598-017-10390-2

**Published:** 2017-09-08

**Authors:** Thomas Knöpfel, Eva M. Pastor-Arroyo, Udo Schnitzbauer, Denise V. Kratschmar, Alex Odermatt, Giovanni Pellegrini, Nati Hernando, Carsten A. Wagner

**Affiliations:** 1 Institute of Physiology, University of Zurich, Zurich, Switzerland; 2 Division of Molecular and Systems Toxicology, Department of Pharmaceutical Sciences, University of Basel, Basel, Switzerland; 3 National Center for Competence in Research NCCR Kidney.CH, Zurich, Switzerland; 4 Laboratory for Animal Model Pathology (LAMP), University of Zurich, Zurich, Switzerland

## Abstract

NaPi-IIb/Slc34a2 is a Na^+^-dependent phosphate transporter that accounts for the majority of active phosphate transport into intestinal epithelial cells. Its abundance is regulated by dietary phosphate, being high during dietary phosphate restriction. Intestinal ablation of NaPi-IIb in mice leads to increased fecal excretion of phosphate, which is compensated by enhanced renal reabsorption. Here we compared the adaptation to dietary phosphate of wild type (WT) and NaPi-IIb^−/−^ mice. High phosphate diet (HPD) increased fecal and urinary excretion of phosphate in both groups, though NaPi-IIb^−/−^ mice still showed lower urinary excretion than WT. In both genotypes low dietary phosphate (LDP) resulted in reduced fecal excretion and almost undetectable urinary excretion of phosphate. Consistently, the expression of renal cotransporters after prolonged LDP was similar in both groups. Plasma phosphate declined more rapidly in NaPi-IIb^−/−^ mice upon LDP, though both genotypes had comparable levels of 1,25(OH)_2_vitamin D_3_, parathyroid hormone and fibroblast growth factor 23. Instead, NaPi-IIb^−/−^ mice fed LDP had exacerbated hypercalciuria, higher urinary excretion of corticosterone and deoxypyridinoline, lower bone mineral density and higher number of osteoclasts. These data suggest that during dietary phosphate restriction NaPi-IIb-mediated intestinal absorption prevents excessive demineralization of bone as an alternative source of phosphate.

## Introduction

Phosphate (Pi) is vital for many biological functions including energy metabolism, intracellular signaling, structural composition of cellular membranes, and bone mineralization. Pi homeostasis is regulated by the coordinated interplay of different organs and endocrine networks. The intestine absorbs Pi from the diet and kidneys reabsorb Pi from the primary urine filtrate. Additionally, the bones serve as a reservoir for Pi, where it can be deposited as hydroxyapatite or released in case Pi supply is low^1, 2^. Under normal conditions, osteoblastic bone formation is in balance with osteoclastic bone resorption. Several hormones such as parathyroid hormone (PTH) or glucocorticoids can stimulate osteoclast activity and thereby increase bone mineral release and promote demineralization^3, 4^.

The Slc34 family of Na^+^-dependent Pi transporters plays an essential role in Pi homeostasis. In the murine intestine, transcellular transport of Pi occurs mainly in the ileum, where NaPi-IIb (Slc34a2) is localized^5, 6^. NaPi-IIb seems to be the major murine intestinal Pi transporter, as its depletion results in abrogation of Na^+^-dependent transport of Pi and increased fecal loss of Pi^7, 8^. Although mutations in NaPi-IIb have been described as a main cause of pulmonary alveolar microlithiasis in humans^9^, Pi metabolism in most of these patients has not been investigated. NaPi-IIa (Slc34a1) and NaPi-IIc (Slc34a3) are responsible for the renal reabsorption of Pi. Both transporters are localized in the brush border membrane (BBM) of the renal proximal tubular cells^10, 11^. NaPi-IIa accounts for the majority of Pi reabsorption, since its ablation in mice leads to severe renal Pi wasting and hypophosphatemia, resulting in underdeveloped bone trabeculae, impaired bone formation and nephrocalcinosis in young mice^12, 13^. Hypophosphatemia and nephrocalcinosis have been also reported in patients with mutations in NaPi-IIa^14, 15^, and gene wide association studies indicate a strong correlation between NaPi-IIa and plasma levels of Pi^16^. In contrast, deletion of NaPi-IIc does not impair Pi homeostasis in mice^17, 18^; however, many studies have reported mutations in NaPi-IIc in patients with hereditary hypophosphatemic rickets with hypercalciuria (for review see refs 19 and 20). The Slc20 family of Na^+^-dependent Pi transporters consists of Pit1 and Pit2, both showing broad tissue distribution including the epithelia of intestine and the renal proximal tubule^21^. Their contribution to intestinal and renal transport of Pi remains to be tested, though the renal expression of Pit2 is regulated by factors controlling Pi homeostasis^22^.

The abundance of Slc34 transporters is regulated by a hormonal network consisting of PTH, fibroblast growth factor 23 (FGF23) and vitamin D_3_ (for review see refs 23 and 24). PTH and FGF23 target the kidney to promote phosphaturia by removing NaPi-IIa and NaPi-IIc from the BBM of proximal cells (for review see ref. 25), whereas vitamin D_3_ acts on the intestine to stimulate absorption of Pi by increasing the expression of NaPi-IIb^26^. In addition, each of these hormones controls the levels of the other two (for review see refs 23 and 24).

The dietary Pi content influences the levels of Pi-regulating hormones and Pi-transporters. Thus, low dietary Pi as well as hypophosphatemia increase the abundance of NaPi-IIa, NaPi-IIc and NaPi-IIb^22, 27–30^ and thereby promote intestinal absorption and renal reabsorption. These changes are at least in part secondary to higher plasma levels of vitamin D_3_ and reduced concentrations of FGF23 and PTH^28^. However, studies in VDR deficient mice indicate that stimulation of NaPi-IIb also occurs in a vitamin D_3_ independent fashion^29, 30^. The reduced levels of FGF23 and PTH triggered by hypophosphatemia remove the suppression of renal NaPi-IIa and NaPi-IIc and thereby enhance renal Pi reabsorption. In contrast, high dietary Pi as well as hyperphosphatemia stimulate PTH and FGF23 and reduce vitamin D_3_^28, 31, 32^. In addition to its phosphaturic effect, high FGF23 also reduces the production of vitamin D_3_ and increases its degradation^33^, blunting the stimulatory effect on intestinal Pi absorption. Besides their effects on Pi handling, vitamin D_3_ and PTH also control the levels of plasma Ca^2+^ by stimulating the expression of intestinal (TRPV6, calbindin D_9K_ and Ca^2+^-ATPase) and renal (TRPV5, calbindin D_28K_ and NCX1) proteins involved in epithelial Ca^2+^ transport (for review see refs 34 and 35) as well as by stimulation of osteoclasts and thereby bone resorption^36^.

As indicated above, intestinal ablation of NaPi-IIb in mice abolishes Na^+^-dependent Pi uptake into ileal BBM^7, 8^. However, under standard dietary conditions these mice only show slightly increased fecal Pi loss, which is compensated by reduced urinary excretion, thus preserving normophosphatemia. Since the expression of NaPi-IIb is upregulated by low dietary Pi^37^, the role of NaPi-IIb may become more important once dietary Pi is restricted. In this study we compare the effect of the dietary Pi content, particularly Pi-restriction, in wild type (WT) and intestinal-specific NaPi-IIb deficient mice (NaPi-IIb^−/−^) and show that upon Pi-restriction NaPi-IIb^−/−^ mice demineralize bone which may help to prevent more severe hypophosphatemia.

## Results

### Intestinal ablation of NaPi-IIb and Pi deprivation causes transient hypophosphatemia and exacerbates urinary calcium excretion

**Figure 1:**
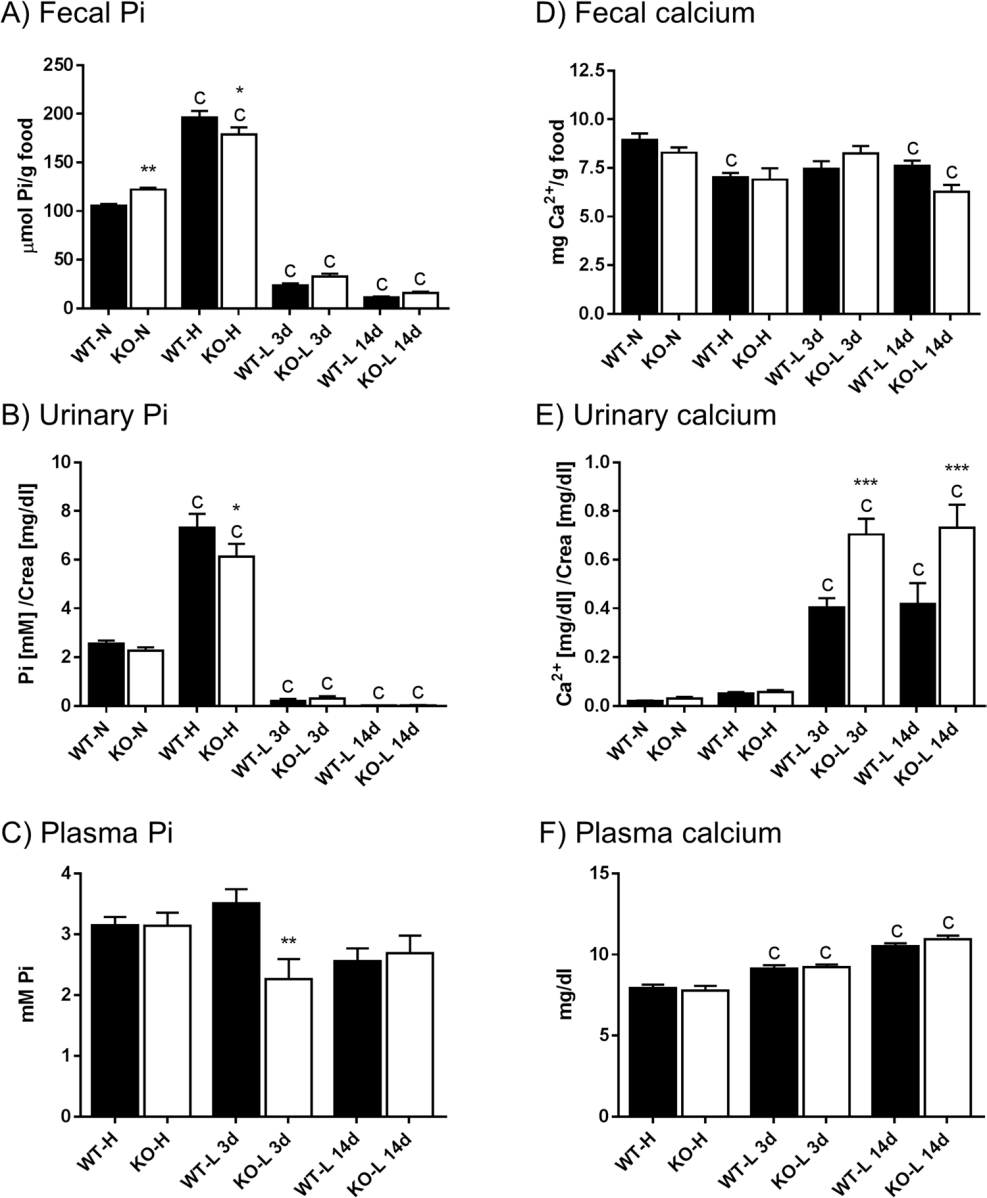
Intestinal ablation of NaPi-IIb and Pi deprivation causes transient hypophosphatemia and stimulates urinary calcium excretion. Fecal (**A**), urinary (**B**) and plasma (**C**) concentrations of Pi as well as fecal (**D**), urinary (**E**) and plasma (**F**) levels of Ca^2+^ were measured in samples collected from wild type (WT) and NaPi-IIb^−/−^ mice (KO). Mice were fed diets containing normal (**N**), high (**H**) or low (**L**) amounts of Pi. The high Pi diet was provided for 3 days (3d) whereas the low Pi diet was provided for 3 (3d) and 14 days (14d), respectively. Data is presented as mean + SEM (n = 10) and was analyzed by ANOVA-Bonferroni. Significant differences are indicated as: ^a^/*p < 0.05, ^b^/**p < 0.01 and ^c^/***p < 0.001, where letters indicate significant changes versus normal diets (or versus the high Pi diet, if normal diet is not available), and asterisks mark differences between genotypes under the same dietary condition.

Urinary and stool samples from WT and NaPi-IIb^−/−^ mice were collected in metabolic cages under standard conditions as well as after adaptation to either high dietary Pi (HPD) for 3 days, or to low dietary Pi (LPD) for 3 or 14 days (Fig. 1). For both genotypes, the fecal excretion of Pi reflected the dietary Pi content: it was higher in animals fed high Pi and progressively lower in those fed low Pi as compared with mice kept on normal chow (Fig. 1A). Except for the HPD, there was a tendency for increased fecal Pi excretion in NaPi-IIb^−/−^ compared with WT mice, which was only significant during normal dietary conditions when absolute values were compared. However, the relative differences in fecal Pi excretion between both genotypes actually increased with decreasing levels of Pi in the diet: NaPi-IIb^−/−^ showed around 13% higher excretion than WT mice when fed normal diet whereas the increase was 41% and 45% in the groups fed low Pi for 3 and 14 days, respectively (supplementary Figure 1).

In both genotypes, the urinary excretion of Pi also adapted to the dietary Pi content, being higher in mice fed HPD diet and lower in the groups fed LPD as compared with mice receiving standard food (Fig. 1B). NaPi-IIb^−/−^ mice excreted significantly less Pi in urine than the WT littermates after 3 days of HPD adaptation. Pi excretion was almost undetectable after 3 or 14 days of LPD, and no differences between genotypes were observed.

The different dietary conditions did not result in significant changes in plasma Pi values neither in WT nor in NaPi-IIb^−/−^ mice (Fig. 1C). However, due to a transient trend for hypophosphatemia in NaPi-IIb^−/−^ fed LPD, plasma Pi was lower in mutant mice than in WT upon 3 days LPD. No differences between both genotypes were detected in the other dietary conditions.

The fecal excretion of Ca^2+^ was similar in both genotypes regardless of the feeding protocol, though there was a tendency for reduced excretion in animals fed on both experimental diets (Fig. 1D).

In both genotypes urinary Ca^2+^ excretion was comparable in mice fed a normal diet or HPD for 3 days, whereas a drastic increase was observed in animals fed a LPD for either 3 or 14 days (Fig. 1E). This increase was even stronger (more than 70%) in NaPi-IIb^−/−^ compared to WT mice.

Additionally a lower amount of creatinine was excreted in NaPi-IIb^−/−^ mice after long term dietary Pi restriction (Table 1), but the creatinine clearance as an indicator for glomerular filtration rate (GFR) was not significantly altered, and excretion of other measured ions was comparable to WT littermates (Table 2).

**Table 1:** Metabolic data.

	WT-N	KO-N	WT-H	KO-H	WT-L 3d	KO-L 3d	WT-L 14d	KO-L 14d
Body weight [g/24 h]	25.26 ± 0.41	26.17 ± 0.46	23.64 ± 0.29	22.80 ± 0.62^c^	23.23 ± 0.86	23.81 ± 0.78^a^	24.01 ± 0.49	22.89 ± 0.72^c^
Food intake [g/24 h]	4.11 ± 0.18	3.82 ± 0.19	3.14 ± 0.10^a^	2.35 ± 0.21^c^	3.37 ± 0.28	2.97 ± 0.23	3.62 ± 0.11	2.65 ± 0.27^b*^
Water intake [ml/24 h]	4.16 ± 0.17	3.99 ± 0.18	4.43 ± 0.67	4.53 ± 0.54	3.04 ± 0.20	3.93 ± 0.49	3.36 ± 0.23	3.89 ± 0.46
Feces weight [g/24 h]	1.02 ± 0.04	0.94 ± 0.05	0.43 ± 0.02^c^	0.25 ± 0.03^c^	0.43 ± 0.03^c^	0.38 ± 0.04^c^	0.44 ± 0.01^c^	0.30 ± 0.03^c^
Fecal Pi [μmol/g food]	105.7 ± 1.7	122.2 ± 2.0^**^	196.2 ± 6.8^c^	178.6 ± 7.6^c*^	23.5± 2.7^c^	32.7 ± 3.1^c^	11.2 ± 1.0^c^	16.0± 1.2^c^
Urinary volume [ml/24 h]	0.91 ± 0.10	1.37 ± 0.18	0.99 ± 0.12	1.62 ± 0.52	0.54 ± 0.11	1.32 ± 0.33	0.65 ± 0.14	1.47 ± 0.23
Urinary creatinine [mg/dl]	71.74 ± 5.31	55.49 ± 4.28	56.47 ± 9.86	38.63 ± 7.62	66.01 ± 7.16	54.56 ± 11.23	79.77 ± 15.48	40.27 ± 6.69^*^
Total creatinine [mg/24 h]	0.59 ± 0.05	0.66 ± 0.05	0.44 ± 0.05	0.35 ± 0.07^b^	0.31 ± 0.04^b^	0.44 ± 0.06	0.32 ± 0.06^b^	0.49 ± 0.05
Urinary Pi [Pi/Crea]	2.56 ± 0.13	2.27 ± 0.14	7.32 ± 0.57^c^	6.13 ± 0.53^c*^	0.21 ± 0.08^c^	0.31 ± 0.10^c^	0.02 ± 0.00^c^	0.03 ± 0.00^c^
Total urinary Pi [µmol]	150.30 ± 12.38	146.00 ± 13.09	320.50 ± 38.56^c^	223.80 ± 50.68	6.77 ± 2.28^c^	12.50 ± 4.22^c^	0.82 ± 0.17^c^	1.55 ± 0.20^c^
Urinary calcium [Ca^2+^/Crea]	0.020 ± 0.002	0.032 ± 0.006	0.052 ± 0.005	0.057 ± 0.008	0.405 ± 0.038^c^	0.704 ± 0.065^c***^	0.418 ± 0.086^c^	0.732 ± 0.095^c***^
Total urinary calcium [µmol]	1.10 ± 0.09	2.24 ± 0.54	2.28 ± 0.18	2.46 ± 0.70	13.53 ± 2.33^b^	34.15 ± 3.36^c***^	17.25 ± 4.35^c^	36.68 ± 5.69^c***^
Plasma Pi [mM]	n.a.	n.a.	3.15 ± 0.14	3.14 ± 0.22	3.51 ± 0.24	2.26 ± 0.33^**^	2.56 ± 0.21	2.69 ± 0.29
Plasma Calcium [mg/dl]	n.a.	n.a.	7.94 ± 0.20	7.78 ± 0.30	9.12 ± 0.22^c^	9.22 ± 0.17^c^	10.50 ± 0.19^c^	10.94 ± 0.23^c^

Additional parameters measured in wild type (WT) and NaPi-IIb^−/−^ mice (KO) fed diets containing normal (N), high (H) and low (L) amounts of Pi. The high Pi diet was provided for 3 days (3d) whereas the low Pi diet was provided for 3 (3d) and 14 days (14d). Data is presented as mean + SEM (n ≥ 10) and was analyzed by ANOVA-Bonferroni. Significant differences are indicated as: ^a^/*p < 0.05, ^b^/**p < 0.01 and ^c^/***p < 0.001, where letters indicate significant changes versus normal diets (or versus the high Pi diet, if normal diet is not available), and asterisks mark differences between genotypes under the same dietary condition.

**Table 2:** Additional urinary parameters measured in samples collected from wild type (WT) and NaPi-IIb^−/−^ mice (KO) after 14 days Pi restriction.

		WT	KO
Creatinine			
clearance	[ml/24 h]	326.00 ± 58.60	462.10 ± 43.97
Na^+^	[µmoles/24 h]	179.60 ± 24.64	180.60 ± 26.99
K^+^	[µmoles/24 h]	155.50 ± 19.65	173.30 ± 20.07
Cl^-^	[µmoles/24 h]	213.80 ± 27.94	208.90 ± 28.58
Mg^2+^	[µmoles/24 h]	124.70 ± 16.10	141.40 ± 16.82

Data is presented as mean + SEM (n ≥ 10) and was analyzed by unpaired t-test.

Plasma levels of Ca^2+^ progressively increased in the groups fed LPD compared with HPD (Fig. 1F). However, there were no differences between WT and NaPi-IIb^−/−^ mice under any dietary condition.

### Hormonal adaptation to dietary Pi is similar in both genotypes

**Figure 2:**
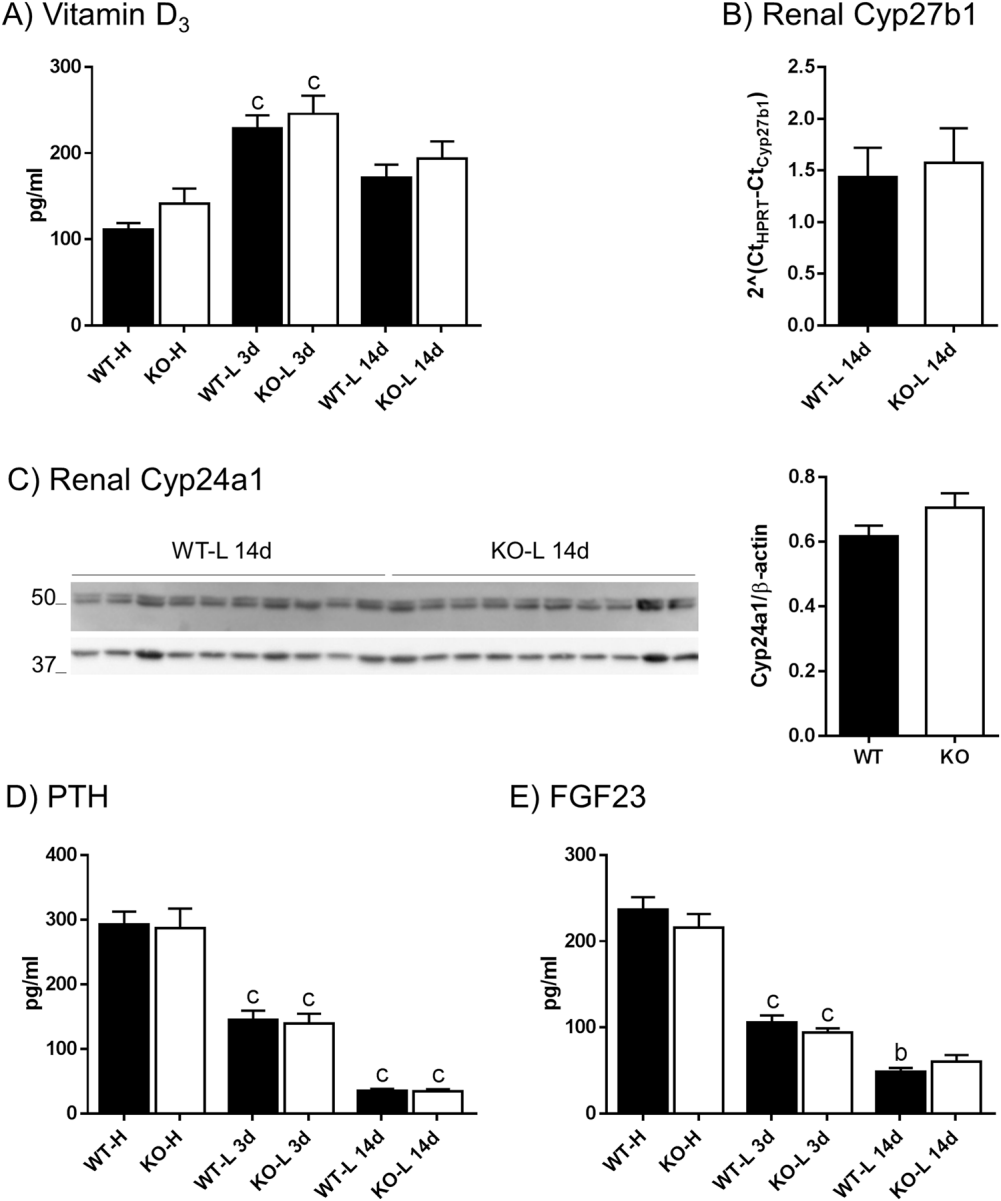
Hormonal adaptation to dietary Pi is similar in both genotypes. Circulating levels of 1,25-(OH)_2_ vitamin D_3_ **(A)**, PTH **(D)** and intact FGF23 **(E)** were measured in plasma collected from wild type (WT) and NaPi-IIb^−/−^ mice (KO) fed diets containing high (H) or low (L) amounts of Pi. The high Pi diet was provided for 3 days (3d) whereas the low Pi diet was provided for 3 (3d) and 14 days (14d). Renal mRNA levels of Cyp27b1 **(B)** and renal protein abundance of Cyp24a1 **(C)** were measured after 14 days of Pi restriction. Data is presented as mean + SEM (n = 10), and was analyzed by ANOVA-Bonferroni. Significant differences are indicated as: ^b^p < 0.01 and ^c^p < 0.001, were significances refer to high dietary Pi groups (no differences between genotypes were observed).

WT and NaPi-IIb^−/−^ mice fed a LPD for 3 days showed higher levels of plasma 1,25-(OH)_2_ vitamin D_3_ compared to animals fed a HPD (Fig. 2A). However, this difference was not observed in the groups fed LPD for 14 days. 1,25-(OH)_2_ vitamin D_3_ levels were similar in WT and NaPi-IIb^−/−^ mice under all conditions. Renal mRNA expression of the vitamin D_3_ activating enzyme Cyp27b1 and protein abundance of the vitamin D_3_ catabolizing enzyme Cyp24a1 was analyzed in animals fed LPD for 14 days. There were no differences between WT and NaPi-IIb^−/−^ mice neither on Cyp27b1 (Fig. 2B), nor on Cyp24a1 (Fig. 2C).

The concentration of plasma PTH was significantly lower in animals fed a LPD for 3 days than in animals adapted to HPD (Fig. 2D). Long term dietary Pi restriction led to a further decrease in plasma PTH. However, WT and NaPi-IIb^−/−^ mice showed similar PTH levels after adapting to the different dietary conditions. For intact FGF23 levels, a similar pattern to PTH was observed (Fig. 2E), i.e. LPD resulted in a progressive reduction of plasma FGF23. No differences between genotypes were observed under the same conditions.

### Upon Pi restriction, Pi transport into renal BBMVs and expression of NaPi-IIa and NaPi-IIc is similar in both genotypes

**Figure 3:**
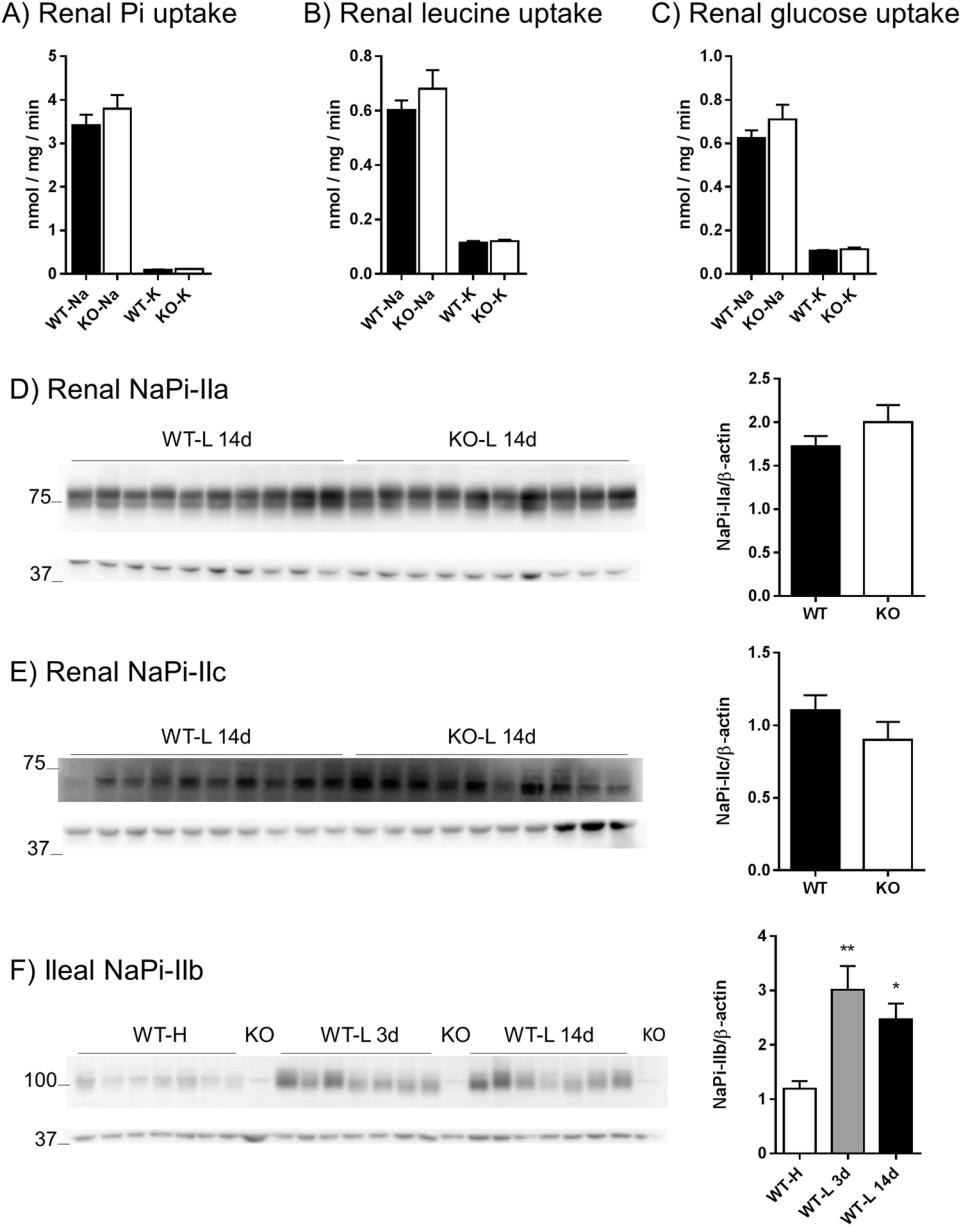
Upon Pi restriction, Pi transport into renal BBMVs and expression of NaPi-IIa and NaPi-IIc is similar in both genotypes. Uptakes of Pi (**A**), leucine (**B**) and glucose (**C**) were performed with renal BBMVs isolated from wild type (WT) and NaPi-IIb^−/−^ mice (KO) after 14 days of dietary Pi restriction (n = 10). Experiments were carried out in the presence (Na) and absence (K) of Na^+^. The renal expression of NaPi-IIa (**D**) and NaPi-IIc (**E**) was quantified by Western blot of the same BBMV used for the uptake experiments; the bar graphs show the corresponding densitometric analysis normalized for the expression of β-actin (n = 10). The abundance of NaPi-IIb in ileum (**F**) was quantified by Western blot in samples from wild type mice (WT) fed 3 days high (H) or low Pi (L) diets as well as 14 days low Pi (n = 7); one sample of a corresponding dietary-matched NaPi-IIb^−/−^ mice (KO) was also included; the bar graph show the densitometric analysis normalized for the expression of β-actin. Uptakes and densitometry values are shown as mean + SEM. Differences between genotypes were analyzed by unpaired t-test (**A–E**), whereas differences versus the high dietary group were analyzed by ANOVA-Bonferroni (**F**). Significant differences are indicated as *p < 0.05 and **p < 0.01.

Transport studies were performed in renal BBMVs from mice fed LPD diet for 14 days. Uptakes were done in presence and absence of sodium, to determine sodium dependent and independent components of Pi, leucine and glucose transport. Uptakes of leucine and glucose were included as negative controls. Both WT and NaPi-IIb^−/−^ mice showed at least a 97% decrease of Pi uptake into renal BBMVs in the absence of sodium, but the sodium dependent and independent transport rates were similar in both genotypes (Fig. 3A). Transport rates of leucine (Fig. 3B) and glucose (Fig. 3C) also showed a strong dependency on sodium (more than 80%), and were similar in both groups of mice.

The protein abundance of NaPi-IIa and NaPi-IIc was assessed in the same renal BBMV preparations that were used for the uptake experiments (long term dietary Pi restriction). In agreement with the urinary Pi and renal Pi-transport data, upon prolonged dietary Pi restriction, the expression of NaPi-IIa (Fig. 3D) and NaPi-IIc (Fig. 3E) were similar in WT and NaPi-IIb^−/−^ mice.

As expected and previously published^37^, NaPi-IIb protein abundance in ileum of WT mice was increased after 3 and 14 days of LPD compared to HPD, whereas the cotransporter was not detected in NaPi-IIb^−/−^ animals (Fig. 3F).

### Upon Pi restriction, the expression of proteins involved in renal calcium handling is comparable in both genotypes

**Figure 4:**
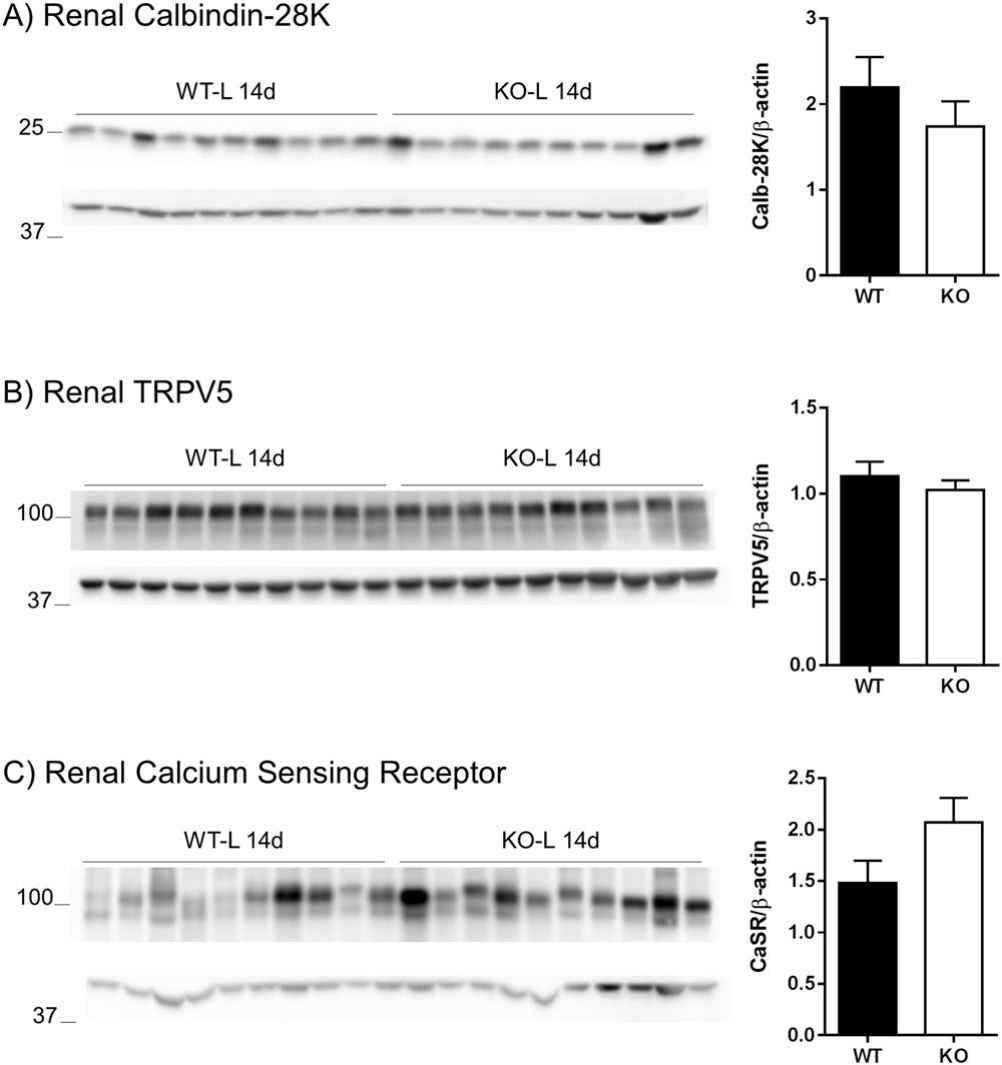
Upon Pi restriction, the expression of proteins involved in renal calcium handling is similar in both genotypes. Renal protein abundance of calbindin-28K **(A)**, TRPV5 **(B)** and calcium sensing receptor **(C)** was assessed by Western blot in homogenates (**A, C**) or BBM (**B**) from kidneys of wild type (WT) and NaPi-IIb^−/−^ animals (KO) after 14 days of Pi restriction. Corresponding densitometric analysis was normalized by the expression level of β-actin and data is shown as mean + SEM (n = 10). Differences between genotypes were analyzed by unpaired t-test.

The abundance of proteins involved in renal Ca^2+^ handling was assessed in WT and NaPi-IIb^−/−^ animals after 14 days dietary Pi restriction. The expression of ﻿﻿﻿calbindin-28K (Fig. 4A) and the Ca^2+^ sensing receptor (CaSR, Fig. 4C) was quantified in renal homogenates whereas the abundance of﻿﻿ the Ca^2+^ channel TRPV5  (Fig. 4B) was analyzed in apical membranes. Similar levels of all three proteins were observed in WT and NaPi-IIb^−/−^ animals.

### Urinary excretion of deoxypyridinoline and corticosterone, bone mineral density and osteoclast number are altered in Pi-restricted NaPi-IIb^−/−^ mice

**Figure 5:**
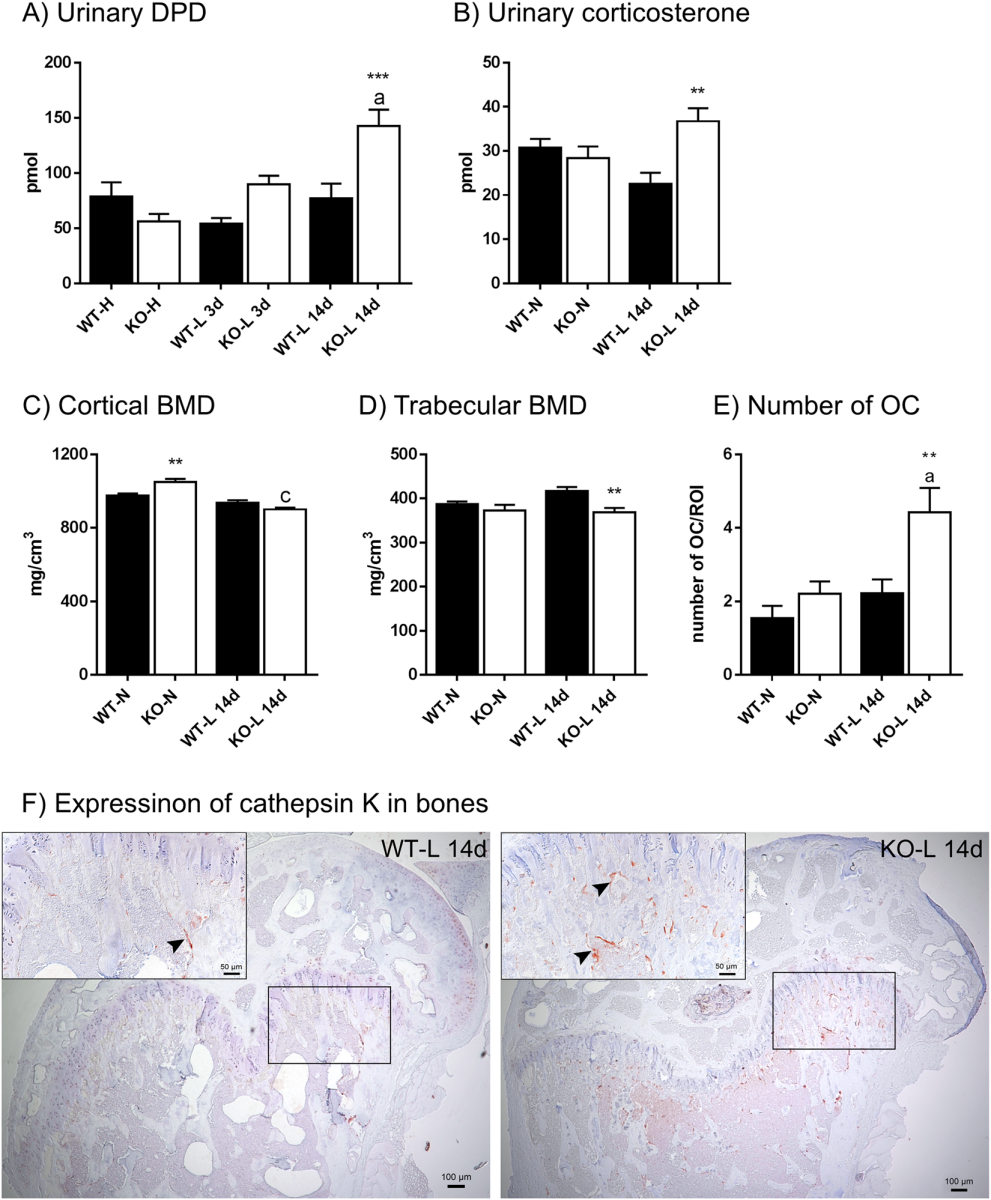
Urinary excretion of deoxypyridinoline (DPD) and corticosterone as well as bone mineral density (BMD) and osteoclast number per tissue area are altered in Pi-restricted NaPi-IIb^−/−^ mice. Urinary DPD (**A**) levels were measured in samples from wild type (WT) and NaPi-IIb^−/−^ mice (KO) mice fed 3 days high (H) or low (L) Pi diets as well as 14 days low Pi. Corticosterone levels in urine (**B**) were determined in samples from mice fed normal diet (N) as well as after 14 days of Pi restriction (L). Cortical (**C**) and trabecular (**D**) BMD were measured in femurs of mice fed normal diet (N) as well as after 14 days Pi restriction. Numbers of osteoclasts expressing cathepsin K (**E**) were assessed in the primary spongiosa of wild type (WT) and NaPi-IIb^−/−^ mice (KO) under normal dietary conditions (N) as well as after 14 day low Pi intake and expressed as average number of cells per 100 μm^2^. Representative pictures of cathepsin K-decorated osteoclasts (red-brown reaction, arrowheads) in the metaphysis of wild type and NaPi-IIb^−/−^ mice after 14 days of Pi restriction are shown in panel F. Data is presented as mean + SEM (n ≥ 7) significance was analyzed by ANOVA-Bonferroni. Significant differences are indicated as: ^a^/*p < 0.05, ^b/^**p < 0.01 and ^c/^***p < 0.001, where letters represent the difference versus the corresponding high dietary group, whereas asterisks mark differences between genotypes under the same dietary conditions.

Urinary excretion of deoxypyridinoline (DPD) was quantified as a marker for bone resorption (Fig. 5A). The dietary content of Pi did not influence the urinary concentration of DPD in WT animals; however, LPD triggered a progressive increase in the amount of DPD excreted into urine by intestinal NaPi-IIb^−/−^ mice. No difference was observed between WT and NaPi-IIb^−/−^ fed a HPD or LPD for three days, whereas after long term Pi restriction the levels of DPD in the urine of NaPi-IIb^−/−^ animals were significantly higher (about 85%) than in the corresponding WT animals.

Urinary corticosterone levels were similar in both genotypes under normal dietary conditions but were elevated in NaPi-IIb^−/−^ mice compared to WT litter mates after 14 days of dietary Pi restriction (Fig. 5B). The corticosterone metabolites 11-dehydrocorticosterone and 5α-dihydrocorticosterone were also increased in NaPi-IIb^−/−^ animals (113.2 ± 19.9 and 176.2 ± 14.8, respectively) compared to WT (52.0 ± 10.4 and 109.0 ± 15.5, respectively) after 14 days LPD.

Bone mineral density (BMD) was measured in femurs of WT and NaPi-IIb^−/−^ mice under normal dietary conditions as well as after 14 days LPD. Cortical BMD was elevated in NaPi-IIb^−/−^ compared to WT under standard diet conditions, whereas trabecular BMD was similar in both groups (Fig. 5C and D). Cortical BMD was significantly reduced in NaPi-IIb^−/−^ mice after dietary Pi restriction, whereas it remained unchanged in WT animals. Trabecular BMD was reduced in NaPi-IIb^−/−^ mice when challenged with long term dietary Pi restriction, compared with the WT littermates (Fig. 5C and D). The number of osteoclasts lining the bony trabeculae in the distal femoral metaphysis of WT and NaPi-IIb^−/−^ mice under normal dietary conditions, as well as after 14 days LPD, was assessed using immunohistochemistry for cathepsin K. While the number of osteoclasts expressing cathepsin K was comparable between WT and NaPi-IIb^−/−^ mice under standard diet conditions (Fig. 5E), the latter exhibited higher osteoclast counts after Pi dietary restriction compared to 14 day-Pi-restricted WT littermates (Fig. 5E and F).

## Discussion

NaPi-IIb is considered to be the major apical Pi transporter in the small intestine since its ablation in mice abolishes Na^+^-dependent uptake of Pi into intestinal sacks and BBMV from ileum^7, 8^, indicating the loss of active transcellular transport of Pi in the small intestine. However, under standard feeding conditions the absence of NaPi-IIb only leads to a moderate fecal wasting of Pi, which is compensated by increased renal reabsorption, thus resulting in normal plasma Pi. This mild phenotype suggests an alternative transport pathway across the intestinal epithelium able to supply enough Pi, at least under normal dietary Pi. Passive absorption through the paracellular pathway may contribute to this process when a sufficient gradient of Pi is established across the epithelium. However, in response to low dietary Pi the expression of NaPi-IIb increases^27, 28^, indicating that the active component may be required to adapt to a reduced oral supply of Pi. Therefore, here we investigated whether the contribution of NaPi-IIb becomes higher once dietary Pi is limited.

As observed in WT animals, the fecal excretion of Pi in NaPi-IIb^−/−^ also increased with HPD and decreased under LPD. However, under all dietary conditions ﻿except for the HPD, the fecal Pi output was higher in NaPi-IIb^−/−^ than WT, though the difference between genotypes was significant only in normal diet when ANOVA was applied to the absolute values. The large differences in the fecal Pi content between the HPD and LPD groups may have masked the relatively smaller differences between genotypes in the LPD; indeed, significant differences were observed when the excretion of NaPi-IIb^−/−^ mice was normalized to the excretion of the dietary-matched WT. Moreover, normalization also indicated that the absence of NaPi-IIb had a larger impact in the Pi-restricted groups than in animals fed high Pi. This may indicate that the contribution of the active transport component (mediated mostly by NaPi-IIb) becomes bigger when dietary Pi is low: dietary restriction could theoretically result in lower Pi levels in the intestinal lumen, thus providing a lower gradient for passive transport of Pi across the epithelium. As expected, 1,25-(OH)_2_ vitamin D_3_, a major stimulus for intestinal Pi absorption^26^, was increased in animals fed the LPD compared to HPD. However, no differences were detected between NaPi-IIb^−/−^ and WT mice. This observation was further supported by the finding that the expression of Cyp27b1 and Cyp24a1 (mRNA and protein, respectively) was similar in both groups of mice. These renal enzymes are crucial in controlling the systemic levels of 1,25-(OH)_2_ vitamin D_3_, since Cyp27b1 converts 25(OH)D_3_ into the active 1,25(OH)_2_D_3_, whereas Cyp24a1 catabolizes 1,25-(OH)_2_ vitamin D_3_^38^. The higher NaPi-IIb abundance triggered by low dietary Pi/high 1,25-(OH)_2_ vitamin D_3_ might further increase the relative difference in Pi transport between WT and NaPi-IIb^−/−^ animals, which is in agreement with the observed trend of higher relative differences in Pi excretion as dietary Pi is restricted.

Dietary loading with Pi triggers a phosphaturic response whereas urinary Pi is low upon dietary Pi restriction. These changes inversely correlate with the abundance of the renal transporters NaPi-IIa and NaPi-IIc, which in turn are controlled by the plasma levels of PTH, FGF23, and 1,25(OH)_2_ vitamin D_3_. Thus, dietary supply of Pi increases the abundance of both hormones whereas Pi deficiency reduces their levels^28, 32^. Similarly to the fecal output, also the urinary excretion of Pi adapted in a similar fashion in NaPi-IIb^−/−^ and WT mice, increasing in response to HDP and decreasing upon dietary Pi restriction. Although the increase in renal Pi excretion triggered by the HPD was lower in NaPi-IIb^−/−^ compared to the WT mice, the massive phosphaturia also observed in NaPi-IIb^−/−^ mice indicates that the component responsible for the passive intestinal transport of Pi is able to absorb a considerable amount of Pi which must be later excreted by the kidney. Instead, both genotypes excreted similarly low amounts of Pi after adaptation to LPD. Since WT animals fed low Pi already increased renal Pi reabsorption to a point where almost no Pi is excreted with urine (especially after 14 days LPD), it is not surprising that the kidneys of NaPi-IIb^−/−^ mice cannot further compensate for the impaired intestinal absorption. In agreement with this maximal and similar reduction of urinary Pi excretion the levels of PTH and FGF23, both phosphaturic hormones, were strongly and similarly reduced in WT and NaPi-IIb^−/−^ mice fed LPD. Furthermore, transport of Pi into renal BBMV and protein expression of NaPi-IIa and NaPi-IIc was also similar in WT and NaPi-IIb^−/−^ mice fed LPD during 14 days. The combination of increased fecal loss but similar (low) urinary excretion of Pi detected in NaPi-IIb^−/−^ on LPD likely caused the more pronounced reduction in plasma Pi levels in NaPi-IIb^−/−^ animals after 3 days of LPD. In contrast, WT mice were able to maintain normal Pi values though a tendency for reduced plasma Pi was detected after 14 days of LPD. The pronounced and earlier fall in plasma Pi may indicate that NaPi-IIb^−/−^ animals need an extra-renal compensatory mechanism when facing longer Pi restriction and that this mechanism is activated within a few days after the onset of Pi depletion.

Chronic hypophosphatemia may lead to osteomalacia and hypophosphatemic rickets^39^, due to the mobilization of Pi from bone and/or decreased mineralization of newly formed bone. The stronger increase in urinary Ca^2+^ excretion in the NaPi-IIb^−/−^ mice during LPD suggested bones as source for Pi: although a drastic  hypercalciuria was observed in both genotypes when fed a LPD (both 3 and 14 days), urinary Ca^2+^ levels were more than 70% higher in NaPi-IIb^−/−^ mice than in their WT littermates. High 1,25(OH)_2_ vitamin D_3_ stimulates not only Pi but also Ca^2+^ absorption in the intestine^40^, and therefore it may contribute to the increase in plasma and urinary Ca^2+^ observed in both animal groups with prolonged Pi deprivation. Moreover, intestinal Ca^2+^ absorption may be enhanced by the absence of Pi reacting with free Ca^2+^ in the intestinal lumen. In addition, low PTH may lead to urinary wasting of Ca^2+^, as a main function of this hormone is to promote Ca^2+^ reabsorption in the kidney^41, 42^. However, none of these factors can explain the difference in urinary Ca^2+^ between WT and NaPi-IIb^−/−^ mice, since they were equally affected in both genotypes. Moreover, the renal expression of several proteins involved in Ca^2+^ transport, including TRPV5, calbindin-28K and the CaSR, was also similar in WT and NaPi-IIb^−/−^ mice. Thus, in response to hypophosphatemia, bone resorption may be enhanced in NaPi-IIb^−/−^ mice releasing Pi, together with Ca^2+^, at the cost of bone mass loss. In turn, renal excretion of Ca^2+^ will be higher to remove excessive Ca^2+^. This interpretation is further supported by the changes in urinary excretion of DPD, a marker for bone resorption^43^. DPD excretion was massively elevated in NaPi-IIb^−/−^ mice upon prolonged Pi restriction, whereas no indication for enhanced bone resorption was detected in dietary-matched WT. In agreement with the DPD data, BMD was decreased in Pi-restricted NaPi-IIb^−/−^ mice.

NaPi-IIb^−/−^ mice fed low Pi also showed higher urinary corticosterone, which is accepted to reflect its circulating levels. Glucocorticoids reduce bone formation rates^44^ and increase osteoclast number, promoting bone resorption^45^. The increase in glucocorticoids might partially explain the reduced bone mineralization as well as the higher osteoclast number observed in NaPi-IIb^−/−^ deficient animals. Together, these observations strongly suggest that the compensation for the loss of active intestinal absorption of Pi requires the mobilization of Pi from bones when the dietary supply is low.

In summary, we found that in response to dietary Pi restriction, renal compensation is not sufficient to maintain plasma Pi levels in mice lacking intestinal NaPi-IIb. Instead, and unlike to WT mice, NaPi-IIb^−/−^ develop a transient hypophosphatemia followed by a compensatory mechanism that involves release of Pi from bone and may be mediated by glucocorticoids. Thus, active intestinal Pi absorption mediated by NaPi-IIb is critical to protect bone during periods of low dietary Pi availability.

## Methods

### Animal handling

Experiments were performed in two months old floxed-Slc34a2 male mice expressing villin-driven Cre recombinase (NaPi-IIb^−/−^) and their wild type litter mates (WT), generated as already described in detail^8^. Until reaching the indicated age, all mice were housed in single ventilated cages in an optimal hygienic husbandry facility. At the beginning of the experiment, mice were transferred to individual metabolic cages (Tecniplast, Buguggiate, Italy) and fed standard diets (0.8% Pi, 1% calcium), to collect basal urinary (under mineral oil) and stool samples. Then, animals were randomized in three groups: two of them received either a low (LPD; 0.1% Pi, 1% calcium) or high (HPD; 1.2% Pi, 1% calcium) Pi diet (Kliba Promivi AG, Switzerland) for 3 days, whereas the third group was fed the LPD for 14 days. Each of the 6 final experimental groups consisted of 10 animals. During the whole procedure mice were fed ad libitum with free access to water. Three days before harvesting samples, animals were placed again in metabolic cages to collect urine and stool. Mice were then anesthetized using ketamine and xylazine. Upon opening the abdominal cavity, blood was collected from the vena cava and centrifuged at 4 °C in heparinized tubes for 7 minutes at 7000 rpm. Plasma, organs and scrapings from mucosa of ileum were snap frozen in liquid nitrogen and stored at −80 °C for further analysis. Urine was centrifuged at 10000 rpm for 10 minutes and stored at −20 °C. Experiments were approved by the local veterinary authority (Veterinäramt Zürich) and performed according to Swiss Animal Welfare laws.

### Plasma, urine and stool parameters

The levels of Pi in stool, urine and plasma were colorimetrically determined according to the Fiske Subbarow method^46^. For determination of fecal Pi content, dried stool was first dissolved in 0.6 M HCl for three days, homogenized and centrifuged as reported^7^. The Jaffe method was used to measure urinary creatinine^47^. Urinary calcium, sodium, potassium, magnesium and chloride as well as plasma calcium and creatinine were measured on a UniCel DxC 800 Synchron Clinical System (Beckman Coulter), a service provided by the Zürich Integrative Rodent Physiology (ZIRP) facility. Calcium in stool was quantified using the QuantiChrom Calcium assay kit (Bio-Assay Systems).

### FGF23, PTH, vitamin D and deoxypyridinoline measurement

The levels of intact FGF23 and PTH in plasma were analyzed by ELISA (Immunotopics International, San Clemente, CA, USA), whereas plasma 1,25 (OH)_2_-Vitamin D_3_ was quantified by radioimmunoassay (Immunodiagnostic System, Frankfurt am Main, Germany). Urinary deoxypyridinoline (DPD) was assessed with an enzymatic immunoassay kit (MicroVue DPD EIA, Quidel Corporation, Athens, USA). All assays were performed according to the manufacturers’ protocol.

### Renal brush border membrane vesicle and homogenate preparation

Brush border membrane vesicles (BBMV) were prepared from frozen kidneys as described before^48^. A polytron (PT 10–35, Kinematica GmbH, Lucerne) was used to homogenize kidneys in a buffer containing (in mM) 300 mannitol, 5 EGTA, 12 Tris-HCl (pH 7.1) and complete mini protease inhibitor cocktail (Roche, Switzerland). An aliquot of the homogenate was frozen and stored at −80 °C for western blot analysis. Upon addition of MgCl_2_ (12 mM final concentration), the remaining homogenate was kept on ice for 15 minutes followed by centrifugation at 4500 rpm for 15 minutes at 4 °C. The resulting supernatant was further centrifuged at 17500 rpm for 30 minutes at 4 °C to collect BBMV. The pellet, containing BBMV was resuspended in a buffer consisting of (in mM) 300 mannitol and 20 HEPES-Tris, pH 7.4.

### Uptake of ^32^P-phosphate, ^3^H-D-glucose and ^14^C-isoleucine into renal BBMV

Uptakes were done according to the reported filtration technique^49^. Freshly prepared BBMV were incubated in solutions containing either 100 mM NaCl or 100 mM KCl, both solutions supplemented with 0.1 mM Pi and ^32^P as tracer. ^3^H-D-glucose and ^14^C-leucine uptakes were measured following the same protocol using solutions containing 0.1 mM D-glucose and 0.1 mM isoleucine together with the indicated radiolabeled tracers. Uptakes were left to proceed for either 30 seconds (^3^H-Glucose and ^14^C-leucine) or 1 minute (^32^P). The incorporation of tracers into the BBMV was measured with a β-counter (Packard BioScience). Remaining BBMVs were snap-frozen and stored at −80 °C for western blot analysis.

### Total membrane fractions from ileum

Scrapings from mucosa of ileum were homogenized in a buffer containing (in mM) 200 mannitol, 80 HEPES, 41 KOH and protease inhibitors, pH 7.5. Homogenization was performed with MagNa Lyser Green Beads (Roche), in a Precellys 24 Homogenizer. Upon centrifugation at 800 rpm for 20 minutes at 4 °C, supernatants were further centrifuged at 41,000 rpm for 30 minutes at 4 °C, and pellets containing total membrane proteins were resuspended in the same buffer used for homogenization.

### Western blot

A Bio Rad DC protein assay kit (BioRad, Cressier, Switzerland) was first used to measure the protein concentration of renal homogenates and BBMV as well as of total membrane fractions from ileum. Then, 20 µg of proteins were mixed with Laemmli sample buffer, loaded on 9% or 12% acrylamide SDS-PAGE, and transferred onto polyvinylidene difluoride (PVDF) membranes (Immobilon-P, Millipore, Schaffhausen, Switzerland). Tris buffered saline (TBS) containing 5% fat free powder milk was used to block membranes for 30 minutes at room temperature prior incubation overnight at 4 °C with primary antibodies against NaPi-IIa^10^, NaPi-IIb^6^, NaPi-IIc^50^, TRPV5^51^, CaSR (Thermofisher Scientific), Calbindin D28k (SWANT, Marly, Switzerland), Cyp24a1 (Protein Tech, Manchester, United Kingdom), and β-actin (Sigma-Aldrich, Buchs, Switzerland). Upon 3 washes with TBS, membranes were again blocked and incubated for 2 hours at room temperature with the appropriate (anti-mouse or anti-rabbit) secondary antibody linked to horseradish peroxidase (HRP) (Promega AG, Dübendorf, Switzerland). After 3 washes with TBS, membranes were exposed to HRP substrate (Western Chemiluminescence HRP Substrate, Millipore, Schaffhausen, Switzerland) for 5 minutes. Chemiluminiscence was detected with a LAS-4000 camera system (Fujifilm). Densitometric analysis was performed using ImageJ and the density of the proteins of interest was normalized to β-actin.

### Semi-quantitative real time RT-PCR

Half a kidney was homogenized in RLT buffer supplemented with β-mercaptoethanol. RNA from homogenates was isolated using the Qiagen RNeasy Mini kit (Qiagen, Hombrechtikon, Switzerland) following the protocol provided by the supplier. TaqMan Reverse Transcription Kit (Applied Biosystems, Zug, Switzerland) was then used for reverse transcription of the isolated RNA according to the manufacturers’ protocol. To quantify relative gene expression, specific sets of primers and FAM/TAMRA-labelled probes for Cyp27b1 (Mm01165918_g1,) and Slc34a2 (Microsynth, Switzerland) were used, and their abundance was normalized to the expression of hypoxanthine-guanine phosphoribosyltransferase (HPRT, Microsynth, Switzerland). KAPA PROBE FAST qPCR Kit Master Mix (KAPA BIOSYSTEMS, Boston USA) containing primers (5 µM) and probe (25 µM) was used to amplify cDNA in a 7500 Fast Real Time PCR System (Applied Biosystems, Zug, Switzerland). The cycle number at a given threshold (Ct) was measured and gene expression relative to the expression of HPRT was calculated according to the formula R = 2^(Ct_HPRT_ − Ct_gene of interest_).

### Glucocorticoids

Urinary glucocorticoids were quantified as described in the supplemental information. Full description of the method validation will be published elsewhere. Briefly, to each urine sample (500 μL) internal standards (corticosterone-D_8_ and creatinine-D_3_, 100 µg/mL) were added and samples were diluted to a final volume of 1.9 mL with sodium acetate buffer (100 mM, pH 4.3). For de-conjugation ß-Glucuronidase (10000 units/mL) was added and samples were incubated in a thermoshaker thorough shaking (2 hours, 900 rpm, 55 °C). Samples were centrifuged (10 min, 16,000 × rcf, 4 °C) and supernatants (1800 µL) were used for solid phase extraction (SPE) on Oasis HBL SPE columns. Samples were eluted with methanol (3 × 500 µL), followed by evaporation to dryness and reconstitution in methanol (25 µL, 10 min, 1300 rpm, 4 °C, thermoshaker). The urinary steroids were separated and quantified by ultra-pressure LC-MS/MS (UPLC-MS/MS) using an Agilent 1290 UPLC coupled to an Agilent 6490 triple quadrupole mass spectrometer equipped with a jet-stream electrospray ionization interface (Agilent Technologies, Santa Clara, CA, USA). Analyte separation was achieved using a reverse-phase column (1.7 μm, 2.1 × 150 mm; Acquity UPLC BEH C18; Waters). Masshunter software (Agilent Technologies) was used for data acquisition and analysis.

### Micro Computer Tomography

Freshly isolated femurs were scanned in a Quantum GX microCT Imaging System (PerkinElmer) provided by the ZIRP facility. Distal epiphysis and subsequent diaphysis were imaged for 3 minutes using a 5 mm field of view at a tube current of 100 µA and 90 kV tube voltage. Analyze 12.0 program (AnalyzeDirect, Inc., Overland Park, USA) was used to asses bone mineral density (BMD) and bone volume data. Bones were analyzed from the end of the patellar surface of the distal epiphysis and 100 sections into the diaphysis were averaged. Grey scales of the scans were translated into BMD using a calibration curve obtained by scans of a 1200 mg/cm^3^ hydroxyapatite phantom (Micro-CT HA Phantom, QRM GmbH, Moehrendorf, Germany).

### Histomorphometric analysis

Femoral bones stored in 98% ethanol were washed and decalcified in 10% ethylenediamine tetraacetic acid over a period of 28 days. The bones were trimmed longitudinally, dehydrated through graded alcohols and routinely paraffin wax embedded. Sections (3–5 µm) were cut from the centers of the femurs, mounted on glass slides, deparaffinised in xylene, rehydrated through graded alcohols and stained with hematoxylin and eosin (HE) or subjected to cathepsin K immunohistochemistry (IHC), using standard protocols. IHC was performed using a rabbit anti-cathepsin K antibody (Abcam, United Kingdom). Briefly, sections were deparaffinised in xylene (2 × 5 min) and rehydrated in decreasing concentrations of ethanol (2 × 3 min washes in 100% ethanol, followed by 1 × 3 min wash in 96% ethanol). Sections underwent antigen retrieval by incubation with 10 mM citrate buffer at pH 6.0. Slides were then incubated for 1 h at 37 °C with the primary antisera (1:200, diluted in Dako antibody diluent, Dako-Agilent Technologies, Denmark), followed by incubation for 30 min with a horse radish peroxidase (HRP)-labeled polymer, conjugated to a secondary anti-rabbit antibody (Dako EnvisionTM System, Dako-Agilent Technologies). The reaction was visualized using 3-amino-9-ethylcarbazole (AEC) as chromogen for 10 min, followed by light counterstain with hematoxylin, rinsing for 5 min in tap water and dehydration in ascending alcohols, clearing in xylene, coverslipping and mounting. All immunohistological stains were performed using an Autostainer (Dako Autostainer Universal Staining System Model LV-1, Dako-Agilent Technologies).

All slides were scanned using a digital slide scanner (NanoZoomer-XR C12000; Hamamatsu, Japan), and the number of cathepsin K-labelled trabecular osteoclasts was calculated in the digital slides using the Visiopharm Integrator System (VIS, version 4.5.1.324, Visiopharm, Hørsholm, Denmark). Briefly, five circular regions of interest (ROI) with a radius of 100 μm were randomly selected in each bone in a blinded fashion. The regions of interest were placed in the primary spongiosa beneath the growth plate, excluding the cortical bone (modified from^52^). A threshold classification allowed recognition of positive (red-brown) multinucleated cells in each ROI, and the results were expressed as number of positive cells per 100 μm.

### Statistical Analysis

Unpaired student’s t-test or ANOVA with Bonferroni correction for multiple comparisons were used to analyze comparisons. P-values < 0.05 were considered as significant. Data is presented as Mean + SEM.

## Electronic supplementary material

Supplementary information
