# Supplementary material for: The intestinal phosphate transporter NaPi-IIb (Slc34a2) is required to protect bone during dietary phosphate restriction
Source: Sci Rep. 2017 Sep 8;7:11018. doi: 10.1038/s41598-017-10390-2 (PMC5591270; doi:10.1038/s41598-017-10390-2)

**SUPPLEMENTS**

**The intestinal phosphate transporter NaPi-IIb (Slc34a2) is required to protect bone during dietary phosphate restriction**

**Thomas Knöpfel^1,3^, Eva M. Pastor-Arroyo^1,3^, Udo Schnitzbauer^1,3^, Denise V. Kratschmar^2,3^, Alex Odermatt^2,3^, Giovanni Pellegrini^4^ , Nati Hernando^1,3^*, Carsten A. Wagner^1,3^***

^1^Institute of Physiology, University of Zurich, Switzerland, ^2^Division of Molecular and Systems Toxicology, Department of Pharmaceutical Sciences, University of Basel, Switzerland, ^3^National Center for Competence in Research NCCR Kidney.CH, ^4^Laboratory for Animal Model Pathology (LAMP), University of Zurich

**Quantification of steroids and creatinine in mouse 24 hours urinary samples**

**1.1. Chemicals and reagents**

Acetonitrile and formic acid at UHPLC-grade were purchased from Biosolve (Dieuze, France) or Sigma-Aldrich (St. Louis, MO). Distilled water was obtained using a MilliQ water purification system (Millipore, USA). Corticosterone-D_8_ (98% isotopic purity) was purchased from C/D/N Isotopes Inc (Pointe-Claire, Canada). Creatinine, corticosterone, 11-dehydrocorticosterone, 5α -dihydrocorticosterone (5α-DHB), creatinine-D_3_ (99.9% isotopic purity) and all other chemicals were obtained from Sigma-Aldrich (St. Louis, MO) of the highest grade available.

**1.2. Instrumentation and analytical conditions**

*Solid phase extraction:* Extraction was performed by use of a vacuum manifold (Agilent Technologies, California, USA) equipped with Oasis HBL SPE cartridges (60 mg, Waters, Massachusetts; USA). Samples were evaporated to dryness using a Genevac EZ-2 plus centrifugal vacuum evaporator (Genevac, Suffolk, UK).

*Analytical instruments:* Ultra-High performance liquid chromatography-tandem mass spectrometry (UHPLC-MS/MS) using an Agilent 1290 UHPLC instrument equipped with a binary solvent delivery system, an auto sampler (at 4 °C), and a column oven, coupled to an Agilent 6490 triple quadrupole mass spectrometer equipped with a jet stream electrospray ionization interface (AJS-ESI) (Agilent Technologies, Basel, Switzerland) was used for steroids and creatinine quantification.

*Liquid chromatography:* The chromatographic separation was performed on a Waters Acquity UPLC BEH C18, 1.7 µm, 2.1×150 mm, column (Waters, Wexford, Ireland) at column temperature of 50 ± 0.8 ºC for creatinine and 54 ± 0.8 ºC for steroids. The mobile phase was water-acetonitrile-formic acid (80/20/0.1; v/v/v) with a constant flow rate of 0.6 mL/min for creatinine and water-acetonitrile-formic acid (70/30/0.1; v/v/v) with flow rate of 0.5 mL/min for steroids respectively. Creatinine was separated within 2 min, followed by 1 min column wash at 100 % acetonitrile and subsequent column re-equilibration for 1 min. Steroids were separated with 30 % of mobile phase B at a ramping flow rate from 0.5 ml/min to 0.2 ml/min within 0 - 4.8 min, followed by separation at constant flow rate of 0.2 mL/min using a gradient of B (30 – 10%) during 4.8-7 min and 7.5-13 min at 30 % of mobile phase B. Separation was followed by column wash (100% of mobile phase B, 0.5 mL/min) at 15 min onwards and the run was stopped after 18 min, followed by re-equilibration of the column for 3 min. A methanol in water (75/25 v/v) mixture was used as needle and needle-seat flushing solvent for 10 s after sample injection. Samples were stored until analysis in the auto sampler (maintained at 4 °C). The injection volume was 1 µL per creatinine sample and 5 µL for steroids respectively.

*Mass spectrometry:* Characteristic precursor ions and their corresponding product ions for multiple reaction monitoring (MRM) were defined by use of the compound optimizer software module included within the Mass Hunter Workstation software (Agilent Technologies, California, USA). Analytes were quantified using the corresponding mass transitions: *Creatinine: m/z* 114.07→44.1 (29 V, Dwell 100 ms) and *m/z* 114.07🡪42.1 (40 V, Dwell 200 ms); *creatinine*-D_3_ 117.07→47.1 (29 V, Dwell 100 ms) and *m/z* 117.07🡪45.1 (29 V, Dwell 200 ms); corticosterone *m/z* 347.2→329.2 (9 V, Dwell 150 ms) and *m/z* 347.2→121.1 (21 V, Dwell 200 ms); corticosterone-D_8_ *m/z* 355.2→125.1 (25 V, Dwell 100 ms); 11-dehydrocorticosterone *m/z* 345.2→121.1 (21 V, Dwell 300 ms) and *m/z* 345.2→90.9 (60 V, Dwell 200 ms) and 5α-DHB *m/z* 349.2→313 (13 V, Dwell 150 ms) and *m/z* 349.2→104.9 (41 V, Dwell 200 ms). The AJS-ESI source conditions were optimized using the integrated source optimizer tool and set in the positive ion mode as following: Nitrogen gas temperature (290 °C), gas flow (14 l/min), nebulizer (20 psi), sheath gas temperature (350 °C), sheath gas flow (11 l/min), capillary voltage (4000 V), and nozzle voltage (1500 V). (Agilent Technologies, California, USA, B.08.00/Build 8.0.8023.0).

*Data analysis:* The MassHunter Workstation Acquisition software Version B.08.00/Build 8.0.8023.0 and MassHunter Workstation Software Quantitative Analysis Version B.07.01 /Build 7.1.524.0, respectively (Agilent Technologies, California, USA) was used for data acquisition and subsequent data analysis.

*Sample preparation:* Mouse urine samples were centrifuged at 16.1 x g for 30 min at 4°C. To 500 μl of urinary supernatant or calibrator an internal standard solution containing corticosterone-D_8_ and creatinine-D_3_ (100 µg/ml) was added and samples were diluted to a final volume of 1.9 mL with sodium acetate buffer (100 mM, pH 4.3). To each urine sample ß-Glucuronidase from Helix promatia (10 000 units/mL) were added and samples were incubated in a thermoshaker thorough shaking (2 hrs, 900 rpm, 55 °C). Samples were centrifuged (10 min, 16,000 × rcf, 4 °C). For solid phase extraction supernatant of each sample or calibrator (1800 µL) was transferred to Oasis HBL SPE cartridges (preconditioned with methanol and water, 3 mL each). Samples were washed with water (3x1 mL), water/methanol (3x1 mL, 90/10 v/v) and water-methanol-ammonia (1 mL, 60/40/2; v/v/v). Samples were allowed to dry under full vacuum for 5 minutes and eluted with methanol (3x 500 µL). Samples were evaporated to dryness and reconstituted in 25 µL methanol (10 min, 1300 rpm, 4 °C, thermoshaker).

*Chromatographic performance:* Ten-point calibration curves over the range of 0.002 to 0.6 mmol/L for creatinine and 1.9 to 500 nmol/L for corticosterone, 11-dehydro-corticosterone and 5alpha-dihydro-corticosterone were generated by a zero sample and nine calibrators in phosphate buffered saline. The coefficient of determination (R^2^) was 0.99 and at least 75% of all calibrators had to be valid.

**Supplementary Figure 1:** **Fecal Pi excretion in wild type (WT) and NaPi-IIb^-/-^ mice (KO) after normal (N), 3 days high (H), 3 days low (L 3d) and 14 days low (L 14d) dietary Pi**. Values of the KO mice were normalized to the mean of the dietary-matched WT. Data is presented as mean + SEM (n=10), and was analyzed by ANOVA-Bonferroni, with ***p<0.001 versus the dietary-matched WT.

**Supplementary Figure 1**


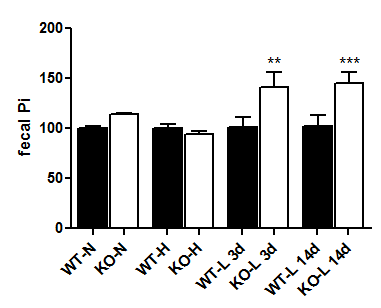

Supplement: Supplementary file 1 — Supplementary information [file 41598_2017_10390_MOESM1_ESM.doc]
